# Comparison of inferior vena cava collapsibility, distensibility, and delta indices at different positive pressure supports and prediction values of indices for intravascular volume status

**DOI:** 10.3906/sag-1810-52

**Published:** 2019-08-08

**Authors:** Aykut SARITAŞ, Çiler ZİNCİRCİOĞLU, Pelin UZUN SARITAŞ, Uğur UZUN, Işıl KÖSE, Nimet ŞENOĞLU

**Affiliations:** 1 Department of Anesthesiology and Reanimation, Health Sciences University Tepecik Training and Research Hospital, İzmir Turkey

**Keywords:** Inferior vena cava, positive pressure, central venouse pressure, intravasculer volume

## Abstract

**Background/aim:**

To compare the inferior vena cava (IVC) indices, identify their variation rates at positive pressure values and accurate predictive values for the volume status in patients with spontaneous respiration receiving different positive pressure support.

**Material and methods:**

The study included 100 patients who were divided into 4 pressure support groups, according to the different pressure supports received, and 3 volume groups according to their CVP values. Ultrasonography was applied to all of the patients to define their IVC diameters at different pressure supports. Dynamic parameters were derived from the ultrasonographic assessment of the IVC diameter [collapsibility (CI-IVC), distensibility (dIVC), and delta (ΔIVC) indices].

**Results:**

There were significant differences between the 3 indices (CI-IVC, dIVC, and ΔIVC) according to the pressure groups [(10/5), (10/0), (0/5), (t tube 0/0)]. The median value for the dIVC percentages was ≤18% for all of the positive pressure support hypervolemic groups, apart from the hypervolemic t tube group (19%). For the hypervolemic groups, the best estimation according to the cut-off value appeared to be for the dIVC. Values with the highest sensitivity for differentiation of the hypovolemic individuals were calculated with the dIVC.

**Conclusion:**

The dIVC had a more accurate predictive role in predicting the volume status when compared with the CI-IVC and ΔIVC, and may be used reliably with positive pressure supports.

## 1. Introduction

For critical patients, determining the intravascular volume status and appropriate fluid management are the most important elements of early targeted treatment [1].

There are many methods used to assess the intravascular volume and for successful fluid resuscitation. Among these monitoring methods, hemodynamic monitoring using ultrasonography (US) remains current due to properties like being noninvasive, ready-to-use, applicable at the bedside, being economic, and being available in most intensive care units [2].

Central venous pressure (CVP) is used to identify the present fluid status and possible fluid requirements, but reliability is reduced due to the invasive nature of the procedure, linked complication risks, and low sensitivity and specificity. As an alternative to this method for assessment of the intravascular volume status among critical patients, the inferior vena cava collapsibility (CI-IVC), distensibility (dIVC), and delta (ΔIVC) indices on dynamic measurements of the IVC diameter have been encountered as more current and increasingly common measurements [3–5]. Measuring the volume status during triggered positive pressure support is necessary to reveal which IVC index is valid. Additionally, the number of studies comparing different positive pressure supports and positive end expiratory pressures (PS/PEEP), IVC indices, prediction of the volume status, and superiority of the indices are limited. 

This study, based on these debates about applications in the relevant literature, aimed to compare the IVC indexes, identify their variation rates at positive pressure values, and correlate with the CVP and accurate predictive values for the volume status of patients with spontaneous respiration receiving different positive pressure support.

## 2. Materials and methods 

This study was approved by the institutional review board and ethics committee of the İzmir Tepecik Training and Research Hospital, Faculty of Medicine Sciences (No: 29/4 18/08/2016), and written informed consent was obtained from each patient’s next of kin. The clinical trial (clinicaltrials.gov) registration number is NCT03452046.

### 2.1. Study design

This prospective observational study was completed in the tertiary intensive care unit at the Tepecik Training and Research Hospital from September 2016 to February 2018. The study included 100 patients. Inclusion criteria for the study were as follows; patients who were >18 years, on a mechanical ventilator with a tidal volume of 6–8 mL/kg, and spontaneous respiration. The main exclusion criteria were as follows: patients who were <18 years, had high intraabdominal pressure findings, had severe right heart failure (tricuspid insufficiency), could not lie in a supine position, had severe tachypnea, whose present peripheral oxygen saturation (SpO2) was <88% without spontaneous respiration, had PS requirements of >16 mmHg and PEEP requirements of >10 mmHg, used high-dose vasopressors, were morbidly obese, and had no clear images obtained via US.

### 2.2. Ultrasound measurements

**Figure 1:**
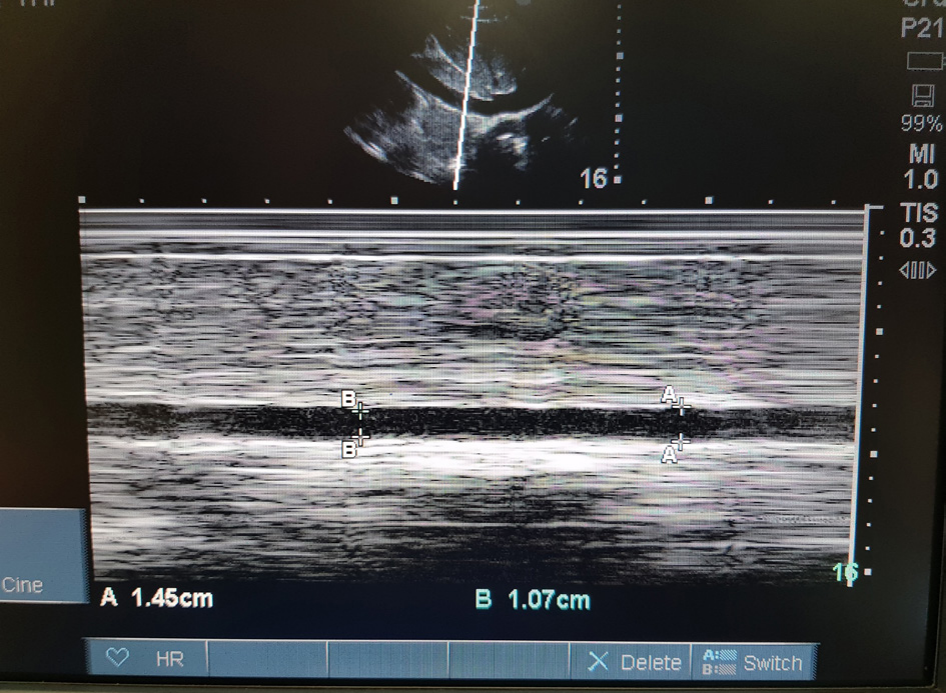
Image of the IVC diameters.

US was applied to all of the patients in a supine position. IVC ultrasonographic measurements were completed with a Sonosite M-Turbo (SonoSite Inc., Bothell, WA, USA) 2–6 mHz phased array probe, longitudinally from the subxiphoidal area, by determining the best localization for imaging, 3–4 cm distal of the IVC-right atrium junction or 2 cm caudal of the IVC-hepatic vein junction (Figure 1). First, the measurement location was identified with 2-D (B mode), and then the M-mode was used for time-motion recording of the IVC. All of the measurements were completed in M-mode, which was also used to capture a 10-s cine loop of the IVC over 2 or 3 respiratory cycles. During the respiratory cycle, the maximum IVC diameter (IVCmax) was measured as the maximum anterior-posterior dimension at the end-expiration and the minimum IVC diameter (IVCmin) was measured at the end-inspiration. The following were calculated: CI-IVC: [(IVCmax – IVCmin) / IVCmax] × 100), dIVC: [(IVCmax – IVCmin) / IVCmin] × 100, and ΔIVC: [(IVCmax – IVCmin) / IVCmedian] × 100). To ensure the interrater reliability, all of the IVC measurements were performed by a clinician with sufficient US education and experience (>50 IVC US evaluations and measurements). All of the recorded videos were investigated by an independent expert.

### 2.3. Ultrasound data collection

After obtaining the location for clear images of the IVC measurement on the US and ensuring probe stabilization, the IVC diameters at different pressure supports were measured by the same clinician. The process of changing the pressure supports was completed by an intensive care doctor blind to the study. In the 5th min after the pressure change, the clinician performing the ultrasonography was allowed to measure, with pressure supports, and the ultrasonographic measurements were recorded by another independent researcher. The pressure support received by the patients was not reported to the clinician measuring the IVC on US to prevent bias during measurements. After the US measurements were complete, the data was combined. Patients were divided into 4 pressure support (PS) groups according to the different pressure supports received:

1. PS 10 mmHg-PEEP 5 mmHg, 

2. PS 0 mmHg-PEEP 5 mmHg 

3. PS 10 mmHg-PEEP 0 mmHg.

4. T tube (PS 0 mmHg-PEEP 0 mmHg)

After each pressure setting, a wait time of 5 min was ensured to obtain optimal sonographic images and for hemodynamics to reach a stable state. Sonographic measurements were performed in the 5th min, after the stable state was reached,

### 2.4. Hemodynamic data collection

Immediately after obtaining the US images at each different pressure setting of the ventilator, an intensive care nurse blind to the study recorded the numerical values of the CVP wave forms in the distal lumen of the central venous catheter. For each CVP wave form measurement, the pressure transducer was set to zero in the midthoracic position. Simultaneous to each CVP measurement, the mean arterial pressure (MAP), heart rate (HR), and SpO2 values were recorded.

### 2.5. Data analysis 

The IVC index cut-off values were taken as a CI-IVC of >50%, dIVC of >18%, and ΔIVC of >12% for the hypovolemia. According to the CVP values, 3 volume groups were distinguished:

1. <8 mmHg hypovolemic 

2. 8–12 mmHg euvolemic 

3. >13 mmHg hypervolemic

 Additionally, for a more detailed assessment of the variations in the CVP with the IVC index percentages, measurements at all positive pressure supports were combined, and from the obtained data (a total of 400 measurements at each pressure variable for 100 patients), the patients were reclassified as:

1. CVP; 0–4 mmHg 

2. 5–9 mmHg 

3. 10–14 mmHg 

4. >15 mmHg 

### 2.6. Statistical analysis

Descriptive information for the participants in the study are presented as n (%), mean, standard deviation, median, minimum, and maximum values.

To check the distribution of the data in the groups, parametric methods were used for the data with normal distribution, while nonparametric methods were used to analyze data with nonnormal distribution. For the dependent multiple group comparisons, the repeated measure ANOVA/Friedman test was used for repeated measurements, while the Dunn test was used for the 2-way comparisons. 

The receiver operating characteristic (ROC) curve analysis was used for the diagnostic cut-off values of indices, with the sensitivity and specificity values calculated. The CVP was taken as the gold standard, with the sensitivity and specificity values of the IVC indices assessed according to the CVP.

When conducting the a priori power analysis, it was calculated such that for the 2-way analysis of variance on repetitive measurements, it was necessary to have a total of 176 measurements (44 measurement in each group), so as to have 80% power for detecting a size effect (f = 0.25) for the partial η2 = 0.06* at P = 0.5 (*: Cohen J, statistical power analysis for the behavioral sciences (revised ed.), 1977).

## 3. Results

### 3.1. Demographic data

The study included 100 patients, and of those, 54% were male and 46% were female. The mean body mass index (BMI) for male patients was 26.06 ± 2.22, while for female patients it was 25.19 ± 25.79. Classification of the patients according to the BMI found that the majority were in the overweight (preobesity) group [6] (Table 1).

**Table 1:** Demographic data.

Variables	Male					Female				
	N	Mean ± SD	Median	Minimum	Maximum	N	Mean ± SD	Median	Minimum	Maximum
Height	54	1.73 ± 0.07	1.73	1.53	1.84	46	1.61 ± 1.6	1.6	1.48	1.8
Weight	54	77.85 ± 9.91	77.5	53	100	46	65.37 ± 66	66	45	83
BMI	54	26.06 ± 2.22	26.06	21.51	31.25	46	25.19 ± 25.79	25.79	20	29.64
Age	54	64.33 ± 15.3	65.5	38	93	46	67.09 ± 71	71	19	93

### 3.2. IVC index and hemodynamic parameters at different positive pressure supports

**Figure 2:**
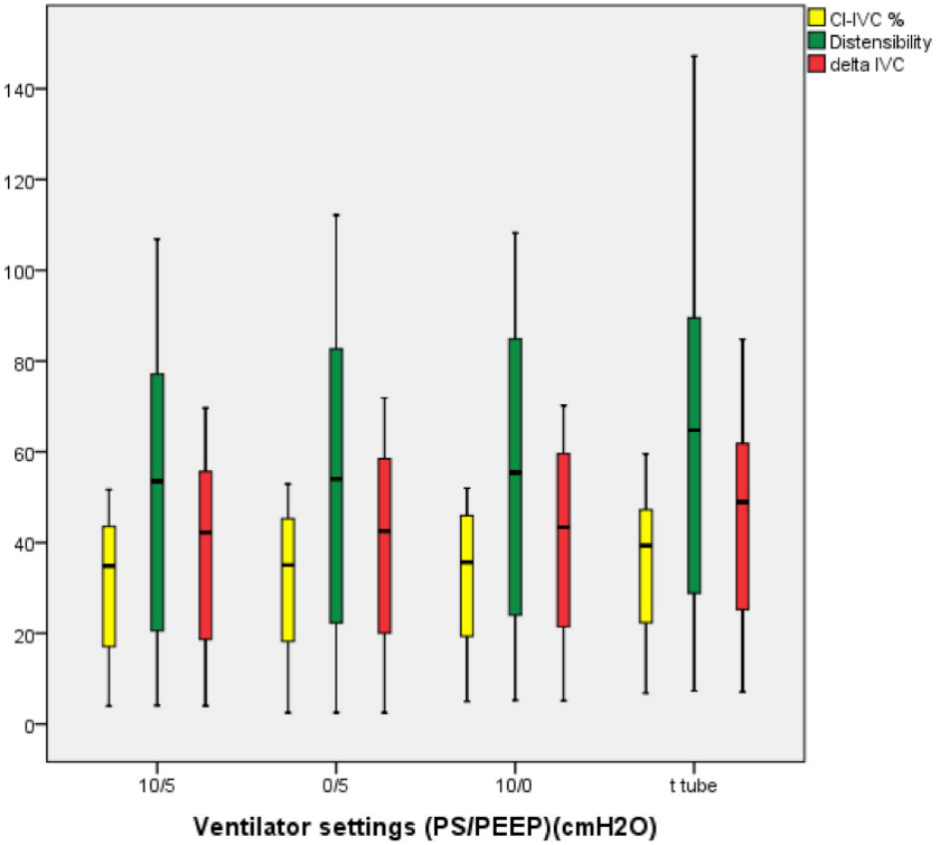
Distribution of the values of the indices at different pressure values.

When the CI-IVC, dIVC, and ΔIVC indices were investigated at different positive pressure supports (PS/PEEP), there were significant differences between the 3 indices according to the pressure groups [(10/5), (10/0), (0/5), (t tube 0/0)] (P < 0.001) (Table 2) (Figure 2).

**Table 2:** Comparison of the variables at different pressure values.

Ventilator settings(PS/PEEP) (mmHg)	10/5	0/5	10/0	t tube	P-value
	Med (min, max)	Med (min, max)	Med (min, max)	Med (min, max)	
Max IVC	18.5 (8.3, 28.2)	18.15 (8.3, 28.4)	17.6 (8.1, 27.8)	16.5 (7.8, 26.5)	<0.001F
Min IVC	11.6 (4.9, 24.4)	11.1(4.7, 27.7)	10.4 (4.6, 25.2)	9.65 (4, 23.9)	<0.001F
CI-IVC	34.8 (3.9, 51.7)	35.05 (2.46, 52.9)	35.7 (4.97, 51.97)	39.3 (6.82, 59.5)	<0.001F
dIVC	53.5 (4.1, 107)	54 (2.5, 112.2)	55.44 (5.23, 108.2)	64.77 (7.32, 147.2)	<0.001F
ΔIVC	42.2 (4.02, 69.7)	42.51(2.5, 71.86)	43.41 (5.1, 70.21)	48.92 (7.06, 84.8)	<0.001F
CVP	6 (–4, 18)	6(–4, 18)	6(–5, 18)	5 (–8, 17)	<0.001F
MAP	76 (43, 110)	77 (45, 112)	76.5 (38, 110)	76 (54, 112)	0.07F
SPO2	96 (90, 100)	96 (80, 100)	96 (81, 100)	96 (88, 100)	<0.001F
HR	88 (58, 120)	88 (60, 121)	86.5 (58, 120)	90 (60, 126)	<0.001F

P < 0.05 significance level. A: ANOVA, F: Friedman test, IVC: inferior vena cava, CI-IVC: collapsibility index, dIVC: distensibility index, ΔIVC: delta index, MAP: mean arterial pressure, CVP: central venous pressure.

Two-way comparisons of each CI-IVC, dIVC, and ΔIVC index, according to the positive pressure groups, identified significant differences (PS/PEEP) [(10/5)–(10/0), (10/5–0/5), (10/5–t tube), (0/5)–(10/0), (0/5)–t tube, (10/0)–t tube].

There were significant differences between different pressure groups in terms of the CVP, SpO2, and HR (P < 0.001); however, there was no statistically significant difference between the groups in terms of the MAP (P = 0.07) (Table 2).

### 3.3. Assessment of the CVP groups for the indices at different pressure supports

When classification was made according to the CVP values (hypovolemic: <8 mmHg, euvolemic: 8–12 mmHg, and hypervolemic: >13 mmHg), the group numbers were reorganized at each different positive pressure support (Table 3).

**Table 3:** Comparison of the indices according to the pressure support and volume groups.

Variables	CI-IVC of 10.5			ΔIVC of 10.5			dIVC of 10.5		
	Hypo	Euvolemic	Hyper	Hypo	Euvolemic	Hyper	Hypo	Euvolemic	Hyper
N	70	11	19	70	11	19	70	11	19
Med (min, max)	41.1(14.15, 2.1)	22.73(9, 42)	12.5 (3.9, 42.6)	52(15.2, 9.6)	25.6(9.4, 53.3)	13.33(4, 69.6)	52 (15.2, 69.7)	25.6(9.4, 53.3)	13.33 (4.02, 54.2)
P-value	<0.001kw			<0.001kw			<0.001kw		
Pairwise comparison	Hypo-euvo(P = 0.175)	Hyper-euvo (P < 0.001)	Hyper-hypo (P = 0.027)	Hypo-euvo(P = 0.175)	Hyper-euvo (P < 0.001)	Hyper-hypo (P = 0.027)	Hypo-euvo(P = 0.185)	Hyper-euvo (P < 0.001)	Hyper-hypo (P < 0.025)
Variables	CI-IVC of 0.5			ΔIVC of 0.5			dIVC of 0.5		
	Hypo	Euvolemic	Hyper	Hypo	Euvolemic	Hyper	Hypo	Euvolemic	Hyper
N	70	12	18	70	12	18	70	12	18
Med(min, max)	42.9(16, 53)	22.3(5.53, 44.9)	12.1(2.46, 20.2)	54.63 (17.8, 72)	25.1 (5.7,57.9)	12.83(2.5, 22.42)	54.63(17.75, 71.86)	25.1(5.69, 57.82)	12.83(2.5, 22.42)
P-value	<0.001kw			<0.001kw			<0.001kw		
Pairwise comparison	Hypo-euvo(P = 0.006)	Hyper-euvo (P = 0.147)	Hyper-hypo (P < 0.001)	Hypo-euvo(P = 0.006)	Hyper-euvo (P = 0.147)	Hyper-hypo (P < 0.001)	Hypo-euvo(P = 0.006)	Hyper-euvo (P = 0.147)	Hyper-hypo (P < 0.001)
Variables	CI-IVC 10.0			ΔIVC of 10.0			dIVC of 10.0		
	Hypo	Euvolemic	Hyper	Hypo	Euvolemic	Hyper	Hypo	Euvolemic	Hyper
N	71	13	16	71	13	16	71	13	16
Med(min, max)	43 (16, 52)	22 (7, 47)	15(5, 28)	56 (17, 70)	24 (7, 62)	16(5,33)	56 (17, 70)	24 (7, 62)	16 (5, 33)
P-value	<0.001kw			<0.001kw			<0.001kw		
Pairwise comparison	Hypo-euvo (P = 0.001)	Hyper-euvo (P < 0.427)	Hyper-hypo (P < 0.001)	Hypo-euvo (P = 0.001)	Hyper-euvo (P < 0.427)	Hyper-hypo (P < 0.001)	Hypo-euvo(P = 0.412)	Hyper-euvo (P < 0.001)	Hyper-hypo (P = 0.001)
Variables	CI-IVC t tube			ΔIVC t tube			dIVC t tube		
	Hypo	Euvolemic	Hyper	Hypo	Euvolemic	Hyper	Hypo	Euvolemic	Hyper
N	72	15	13	72	15	13	72	15	13
Med(min, max)	45 (7, 60)	24 (9, 47)	18 (7, 23)	58(7, 85)	28(9, 62)	19(7, 26)	58 (7, 85)	28 (9, 62)	19 (7, 26)
P-value	<0.001kw			<0.001kw			<0.001kw		
Pairwise comparison	Hypo-euvo (P < 0.001)	Hyper-euvo (P = 0.472)	Hyper-hypo (P < 0.001)	Hypo-euvo (P < 0.001)	Hyper-euvo (P = 0.450)	Hyper-hypo (P < 0.001)	Hypo-euvo(P = 0.001)	Hyper-euvo (P = 0.446)	Hyper-hypo (P < 0.001)

P < 0.05 significance level, kw: Kruskal Wallis test.

When the differences between the volume groups were assessed for each IVC index within each positive pressure support (pressure support groups), significant differences were obtained between the medians within the 95% CIs (P < 0.001). Two-way comparisons between the volume groups and PS groups found statistically significant differences for each of the indices between the hypervolemic and hypovolemic groups (Table 3).

The CI-IVC percentage was <50% in all of the volume and PS groups. In the hypervolemic groups, the median CI-IVC value was significantly low.

The ΔIVC was >12% in all of the volume and PS groups. The median for the hypervolemic groups was significantly low compared to the medians for the hypovolemic and euvolemic groups.

The median value for the dIVC percentages was ≤18% for all of the positive pressure support hypervolemic groups, apart from the hypervolemic t tube group (19%). For the hypervolemic groups, the best estimation according to the cut-off value appeared to be for the dIVC. The median for the hypervolemic groups was significantly low compared to the medians for the hypovolemic and euvolemic groups (Table 3).

### 3.4. Assessment and correlation of the CVP groups with indices according to the total measurement at all positive pressure variables

After the groups were reclassified according to the CVP values, as <4, 5–9, 10—14, and >15, the medians of the indices in the groups were compared. For each of the 3 indices, as the CVP values increased, the indices reduced. Although this numerical decrease was statistically significant in all 3 indices, while the indexes were examined when determining the volume status according to the cut-off values, according to the CVP of >15 group, the most accurate estimation was made from the dIVC index (<18%) (Table 4). 

**Table 4:** Comparison of the indices in the CVP groups.

Variables		N	Mean ± SD	Med (min, max)	P-value	Pairwise comparison
CI-IVC	>0–4	136	39.64 ± 10.88	43.67(6.82, 59.54)	P < 0.001kw	(5–9) & (15+): P < 0.001
	5–9	167	36.85 ± 11.43	41.27 (8.78, 52.17)		(<0–4) & (15+): P < 0.001
	10–14	57	17.1 ± 9.21	16.48 (2.46, 42.96)		(10–14) & (5–9): P < 0.001
	15+	40	12.75 ± 5.43	13.09 (3.94, 23.02)		(<0–4) & (10–14): P < 0.001
dIVC	>0–4	136	70.5 ± 27.44	77.53 (7.32, 147.17)	P < 0.001kw	(5–9) & (15+): P < 0.001
	5–9	167	63.21 ± 27.32	70.27 (9.63, 109.09)		(<0–4) & (15+): P < 0.001
	10–14	57	22.37 ± 16.22	19.74 (2.53, 75.31)		(10–14) & (5–9): P < 0.001
	15+	40	15.05 ± 7.14	15.06 (4.1, 29.9)		(<0–4) & (10–14): P < 0.001
ΔIVC	>0–4	136	50.52 ± 16.07	55.87 (7.06, 84.78)	P < 0.001kw	(5–9) & (15+): P < 0.001
	5–9	167	46.32 ± 16.62	52 (9.18, 70.59)		(<0–4) & (15+): P < 0.001
	10–14	57	19.28 ± 11.8	17.96 (2.5, 54.71)		(10–14) & (5–9): P < 0.001
	15+	40	13.8 ± 6.18	14.01 (4.02, 26.01)		(<0–4) & (10–14): P < 0.001

P < 0.05 significance level, kw: Kruskal Wallis test.

For each index value at the same pressure, the correlation with the CVP values was assessed with high correlation analysis. For all of the pressure groups, the IVC indices had a strong inverse relationship with the CVP values (P < 0.001) (Table 5).

**Table 5:** Correlation of all of the indices (CI-IVC, dIVC, and ΔIVC) with CVP.

Variables	10/5		0/5		10/0		t tube (0/0)	
	ρ	P-value	ρ	P-value	ρ	P-value	ρ	P-value
CVP	1.000	***	1.000	***	1.000	***	1.000	***
Max IVC	0.550**	<0.001	0.554**	<0.001	0.578**	<0.001	0.592**	<0.001
Min IVC	0.732**	<0.001	0.744**	<0.001	0.717**	<0.001	0.720**	<0.001
CI-IVC	–0.669**	<0.001	–0.697**	<0.001	–0.652**	<0.001	–0.629**	<0.001
ΔIVC	–0.653**	<0.001	–0.682**	<0.001	–0.641**	<0.001	–0.623**	<0.001
dIVC	–0.653**	<0.001	–0.682**	<0.001	–0.641**	<0.001	–0.623**	<0.001
OAB	0.587**	<0.001	0.570**	<0.001	0.549**	<0.001	0.531**	<0.001
SPO2	–0.173	0.086	–0.112	0.269	–0.028	0.778	–0.024	0.815
HR	–0.273**	0.006	–0.276**	0.005	–0.291**	0.003	–0.302**	0.002

P < 0.05 significance level, ρ: correlation coefficient.

### 3.5. ROC analysis (sensitivity/specificity)

Assessment of the index values evaluated the sensitivity and specificity values of the other indices using the CVP as the gold standard.

According to the ROC analysis results, values with the highest sensitivity for the differentiation of the hypovolemic individuals were calculated with the dIVC (Table 6). 

**Table 6:** Roc analysis [sensitivity (sens)/specificity (spec)].

Variables	PS 0 PEEP 5			PS 10 PEEP 5			PS 10 PEEP 0			t tube		
	AUC	P-value	Sens/ spec	AUC	P-value	Sens/ spes	AUC	P-value	Sens/ spes	AUC	P-value	Sens/ spes
CI-IVC	0.529	0.704	0.057/1	0.529	0.041	0.57/1	0.55	0.506	0.98/1	0.55	0.506	0.97/1
ΔIVC	0.737	0.002	1/0.47	0.684	0.014	1/0.36	0.656	0.009	1/0.31	0.570	0.424	0.98/0.15
dIVC	0.816	<0.001	1/0.63	0.835	<0.001	0.98/0.68	0.719	<0.001	1/0.43	0.667	0.018	0.98/0.38

## 4. Discussion

In this study, the percentages of the 3 IVC indices varied significantly at each different positive pressure support and it appeared that the dIVC was more effective in the prediction of the volume status when compared to the CI-IVC and ΔIVC.

Olsen et al. determined that positive pressure support in Trendelenburg and reverse Trendelenburg positions changed the CI-IVC, while in the supine position, there was no significant change, although there was a reducing trend observed with PS, PEEP, or a combination of both. This situation was reported to be due to the insufficient sample size (10 healthy volunteers) [4]. In the current study, all of the patients were assessed in the supine position, with variations observed in the max IVC, min IVC, and each IVC index at each different positive pressure support. The IVCmax and IVCmin diameters were determined with the highest at (PS/PEEP) 10/5 mmHg pressure support and the lowest was at t tube (0/0 mmHg). Specifically, in the 0/5 mmHg pressure support group, the IVCmax and IVCmin diameters were higher when compared to the 10/0 mmHg group, leading to the consideration that PEEP support had more effect on the IVC. The largest diameter was observed in the group with PS and PEEP support together. At each different pressure support, although there were significant variations in the IVC indices, the increase that occurred in the t tube group was more obvious. Campodonico et al. reported that increasing the PEEP from 0 to 10 or from 5 to 10 in patients with spontaneous respiration caused a significant reduction in CI-IVC values; however, increasing it from 0 to 5 mmHg did not cause a significant reduction [7]. We thought that the smaller effect on the CI-IVC values at low PEEP levels may have been due to the protocol used in the study. Measurement of the PEEP at 10 mmHg that was completed a short time after the measurements at PEEP 5 mmHg may have caused this. In this study, the measurements were performed after waiting for 5 min between each pressure change, after which hemodynamic stabilization was achieved. Herein, we identified a significant reduction for all of the IVC indices with PEEP and PS support. Stawicki et al., in a study on patients with and without mechanical ventilation support, could not find a statistically significant reduction in the CI-IVC with an increase in the PEEP and reported a significant increase in CVP levels [8].

The results of studies investigating the correlation of the CVP with the sonographic measurements of the IVC diameter are contradictory. Many studies have stated a positive (IVC diameter) or negative (IVC index) correlation between the dynamic measurements of the IVC indices with US and CVP, and stated that it may be reliably used [9–12]. Many previous studies have included patients on mechanical ventilators with spontaneous respiration or deeply sedated. There are very few studies on the correlation between different positive pressure supports and IVC indices. Specifically, studies investigating the relationship between positive pressure ventilation and the dIVC and ΔIVC are very limited.

Baumann et al., in studies researching the correlation between the CI-IVC and internal jugular vein, found a good correlation in patients with spontaneous respiration; however, they determined that there was no statistically significant correlation when positive pressure ventilation was applied [13]. In the current study, for all of the pressure support variables, there was a high negative correlation between the CI-IVC, dIVC, and ΔIVC with the CVP.

Herein, researching the predictive role for the intravascular volume of the IVC indices compared with the CVP, although the CI-IVC values had a statistically significant reduction from hypovolemic group to hypervolemic group, the CI-IVC values were <50% at all of the pressure variables. The 50% cut-off value for the CI-IVC was observed to be insufficient to distinguish the hyper- and hypovolemic status of the patients. There have been many cut-off values defined for the CI-IVC as a marker of fluid responsiveness. Low CVP values (<7 mmHg) are considered to be a good marker of fluid responsiveness [14]. Nagdev et al. stated that a CI-IVC of ≥50% was strongly correlated with low CVP (<8 mmHg) [12]. Contrarily, our study found that the CI-IVC was <50% when the CVP was <8 mmHg. With regards to fluid responsiveness, Müller et al. reported an association with a CI-IVC of >40% , while Corl et al. reported an association with a CI-IVC of ≥25% [15,16]. According to the hypothesis herein, although no cut-off value was determined according to the fluid response, for all of the pressure groups, no patient with a CI-IVC of < 25% was in the hypovolemia group. All of the patients who were euvolemic and/or hypervolemic had a CI-IVC of < 25%, which was in parallel with the study of Corl et al. According to the statistical analysis, when all of the pressure measurements were combined (100 patients and 4 different pressures for 400 measurements), among patients with a CVP ≥10, a CI-IVC of <25% was assessed as fluid unresponsive.

Although there was a statistically significant reduction in the ΔIVC values from the hypovolemic group to the hypervolemic group, all of the pressure values of all of the patients, including the hypervolemic patients, had ΔIVC values of >12%. The ΔIVC cut-off value of 12% was insufficient to distinguish the hyper and hypovolemic status of the patients. According to the statistical analysis, when all of the pressure measurements were combined, in patients with a CVP of ≥10, a ΔIVC of >12% was unsuccessful in predicting fluid responsiveness.

In this study, the dIVC values statistically significantly reduced from the hypovolemic group toward the hypervolemic group and as the CVP increased, the dIVC decreased. The 18% cut-off value for the dIVC was successful in distinguishing the hypervolemic patients. Although the t tube hypervolemic group had a value of 19%, for all of the other positive pressure supports, <18% successfully distinguished the hypervolemic patients. According to the statistical analysis, combining all of the pressure measurements, in patients with a CVP of 10–14, a dIVC of >18% was found (19.74), while for those with a CVP of ≥15, a dIVC of <18% was identified. As the dIVC value for patients with a CVP of 10–14 was close to the 18% cut-off value, it was accepted as successful in distinguishing hyper- and hypovolemia. There was no proximity to the cut-off values identified for the CI-IVC and ΔIVC values. In all of the CVP groups, a CI-IVC of >50% and ΔIVC of >12% were found, and was insufficient to distinguish the volume status according to the cut-off values. The large difference between the dIVC value for patients with a CVP of 5–9 (70.27) and for patients with a CVP of 10–14 (19.74) was noteworthy. This difference and the study results led us to the consideration that the cut-off value for the dIVC may be determined as higher than 18%. Achar et al., in a study of a pediatric population, determined that the dIVC cut-off value for responsive and non-responsive patients was 23.5%, and with the ΔIVC they determined it as 12.2% [5]. Duwat et al. stated that there was a gray zone for dIVC cut-off values (15%–30%) with sensitivity and specificity that was better for values below 15% and above 30% [17]. This topic may be clarified in the future by wider specific studies.

The ideal index will accurately predict volume status, will be sensitive to fluid response, repeatable, easy, and non-invasive. Herein, sensitivity and specificity were calculated to find the index with the most accurate prediction of the intravascular volume status.

For all of the positive pressure variables, the highest value for sensitivity to distinguish the hypovolemic individuals was for the dIVC.

This study had several limitations. First, determination of the volume groups was made only according to the CVP values. Together with the CVP, there was no comparison of the IVC diameter measurements with dynamic preload indices like extravascular lung water, systolic pressure variation, and stroke volume variations. Second, due to the exclusion criteria, the whole intensive care unit population did not participate in the evaluation, with specific results obtained for a restricted population of patients. Third, the correlation of the IVC indices with fluid loading or passive leg raising was not investigated as, they were not part of the study hypothesis.

In critical patients, we think that the dIVC has a more accurate predictive role in predicting volume status when compared with the CI-IVC and ΔIVC, and may be used reliably with positive pressure supports. Additionally, numerical values of the IVC indices are not sufficient to determine the intravascular volume status of critical patients, but should definitely be used with other monitoring methods, and clinical and hemodynamic assessments.
